# Overcoming Systemic Barriers in US Forensic Anthropology Education: Considering Underlying Barriers to Diversity, Equity, Inclusion, and Belonging in Forensic Anthropology

**DOI:** 10.1002/ajpa.70069

**Published:** 2025-06-02

**Authors:** Taylor S. Borgelt, Jesse R. Goliath, Erin B. Waxenbaum

**Affiliations:** ^1^ Department of Anthropology Purdue University West Lafayette Indiana USA; ^2^ Department of Anthropology and Middle Eastern Cultures Mississippi State University Starkville Mississippi USA; ^3^ Cobb Institute of Archaeology, Mississippi State University Starkville Mississippi USA; ^4^ Department of Anthropology Northwestern University Evanston Illinois USA

**Keywords:** belonging, diversity, forensic anthropology education, inclusion

## Abstract

The experience of disparity—relative to race, ethnicity, gender, age, socioeconomic status, disability, sexual orientation, and others—in higher education is deeply connected to legacies of structural harm and stratification. The presence and impact of this harm in education cannot be understated and is important to recognize when considering what our classrooms look like and why. Contending with instances of disparity in higher education and professional spaces extends beyond just valuing diversity in recruitment—gathering a diverse student group and/or workforce without actionable avenues for inclusion and equity in retention (not fostering, supporting, or valuing a tangibly safe space) is a way in which harm and systemic barriers are perpetuated. This commentary applies across disciplines, though this paper deals with specific examples within and discussion of forensic anthropology education within the United States. To work toward the mitigation of systemic barriers and instances of exclusion within forensic anthropology, a number of things need to be addressed, such as (but not limited to) critically engaging with notions of objectivity in theoretical and educational discussions and the performed depersonalization of reflexivity within the subfield. Relevant to the theory around materialization, understanding how these instances and concepts impact how forensic practice is done is important in wanting to ameliorate education for marginalized and/or vulnerable people (students, practitioners, etc.) within the subfield. How the subfield and its central concepts are conceptualized, and then disseminated, matters for the actualization of inclusive spaces and the alleviating of barriers within the discipline.

Over the last several years, there has been ample and increasing discourse around diversity and expanded recruitment across sectors and disciplines (Hu‐DeHart 1993; Dumas‐Hines et al. 2001; Antón et al. 2018; Wagstaff and LaPorte 2018; Yim et al. 2022). In a movement known by many titles—multiculturalism, diversity and inclusion, pluralism—there has been a significant emphasis on establishing diverse, visible representation in spaces that have historically been made less accessible or welcoming (Hu‐DeHart 1993; Dumas‐Hines et al. 2001; Tallman and Bird 2022; Go et al. 2023). While diversity and recruitment are essential considerations in promoting equity, genuine attention to diversity has to extend beyond initial efforts, like recruitment, and perfunctory performance or simple lip service to actionably increase access and equity; the array of people in any given room matters, but it also matters that people feel cared for and made to belong in these learning and professional environments after they have matriculated, thinking about promoting retention and longevity within a community (Goliath et al. 2023).

There are many ways in which harm and exclusion are manifested and fostered within structures of rigid hierarchy, like academia. In conceptualizing harm as pervasive and dynamic, both explicit and subliminal forms of exclusion must be considered (Harrison 1997; Mondesire 2022; Tallman and Bird 2022). If and when diversity, equity, and inclusion are disciplinary and institutional priorities, working to change the fundamental components of theory and practice that instigate and perpetuate harm, exclusion, and othering is essential (Sulé et al. 2022). While this commentary can be applied across disciplines and subject matters, this paper considers how a lack of inclusion and belonging manifests, why it is so engrained, and how these issues can be potentially faced/mitigated within forensic anthropology education. Forensic anthropology is particularly important because of its dual position in both education and practice, as a space that impacts students, educators, professionals, and larger communities. The field bridges education and practice by applying anthropological knowledge to medicolegal contexts and public health issues, aiding in decedent identification, recovery of remains, and influencing not only criminal justice but also human rights investigations and disaster relief efforts (Langley and Tersigni‐Tarrant 2017). The applied and public intersection of culture and forensic practice is obvious and impactful (in people's experiences of living and dying, how communities are recognized and valued, etc.), so it matters that it is critically considered and continually examined.

In thinking about the value of diverse knowledge systems, formal higher education—and the informally transmitted values present within it—it is critical to consider how practice reflects what is taught and facilitates the perpetuation of ideas from the past (Lopez and Jean‐Marie 2021). Theoretical and political stances held, relative to practice, can and do demonstrate personal or biased priorities and values (DeMarrais et al. 1996; Stoetzler and Yuval‐Davis 2002; M'Charek 2013). Thus, the desire to critically look at the core of some systemic issues surrounding a lack of substantial diversity is rooted in interrogating how exclusion is explicitly and implicitly manifested, and how it is imposed onto people experiencing varying vulnerabilities.

Naming and confronting all systemic barriers to diversity present within (forensic) anthropology is an extensive task, and one beyond the scope of this article. The structural and bureaucratic bounds around forensic anthropology practice and forensic anthropology education are different. So, while not an encompassing framework, this paper specifically contends with issues relevant to education. Here, we contribute to and support discourse around dissecting and discussing clear avenues for mitigating known barriers in forensic anthropology education (Pilloud and Passalacqua 2020; Tallman and Bird 2022; Tallman et al. 2022; Goliath et al. 2023; Litavec and Basom 2023; Pink et al. 2025). This paper is focused on presenting and engaging with examples of implicit/discrete ways forensic anthropological, educational spaces are made unwelcoming, hoping to inform future steps and actions toward diversity and equity in recruitment, retention, and the building of the subdiscipline. Identifying potential ways in which exclusionary harm is being communicated to people who experience marginalization (racial, ethnic, gendered, socioeconomic, disability, etc.) is vital to subsequently understanding how to move a given environment forward while prioritizing equity to reduce legacies and realities of harm (Tallman et al. 2022).

## 1 Addressing Significant Barriers to Access

The general issue of access within forensic anthropology (specifically educational and academic spaces) can be, and is already being, addressed in a number of ways, as many practitioners and educators are applying shifts within their work to engage with the dynamic nature of theory and to promote critical thinking among students. This section highlights and outlines ideological and practical shifts that may be beneficial to think about when envisioning and enacting a more equitable educational space. The premises of scientific objectivity and being atheoretical in education and practice serve to perpetuate limited forms of knowledge production. Being able to question and dissect held beliefs, standards, and practices is important for engaging with dynamic perspectives. In signaling this importance, we outline and discuss a few avenues below.

### 1.1 Revising Curricula and Syllabi; Looking at What Is Being Taught and Critically Considering Why

It is important to critically engage with the theoretical history *of* what is taught and why, even if the theory present in the taught material is not explicit. Technical training and scientific knowledge are informed by beliefs and theories about how knowledge is able to be made and by whom (DeMarrais et al. 1996). Utilizing and presenting material that holds exclusionary framing and not holding space for it to be critically examined and/or discussed communicates a scheme of implicit valuation that is limiting and harmful to intellectual curiosity and diverse thought, invalidating people who come to know and experience the work/world in nondominant ways. By prioritizing a singular or dominant perspective through the theory present in learning spaces, other ways of seeing and knowing become implicitly devalued and excluded. This prioritization is enacted through the types of materials and sources used, and is also demonstrated through who is and is not being cited (Edinger 2019). The origins and history of forensic anthropology categorically dismiss and demote the early/foundational contributions of scholars of color and those outside of dominant Western/European‐American contexts (Forensic Anthropology Community 2021; Go et al. 2023). In curricula that foundationally read and cite scholars like Gall, Morton, Hrdlička, Hooton, Dwight, and Stewart (Caspari 2009; Ubelaker 2017), it is important to also name and adequately attend to scholars like Bond Day, Cobb, El‐Najjar, Clark, Warren, Furue, other scholars of color, and scholars outside of the United States and Europe (Go et al. 2023).

We have an obligation to learn from the “right” and “wrong” of the past. We are seeing significant development in the field holistically with greater focus on our history, issues/absence of diversity, as well as an evolution of our theory and method in line with academic and cultural development (e.g., DiGangi and Bethard 2021; Forensic Anthropology Community 2021; Tallman, Kincer, et al. 2021; Tallman, Parr, et al. 2021; Yim et al. 2022; Adams et al. 2023; Go et al. 2023). In particular, the Forensic Anthropology Community (2021, 1) is a website that boasts its goals of “fostering collegiality and furthering efforts to diversify the discipline” of forensic anthropology. It provides a discipline‐specific syllabus with suggestions of topics to cover and associated citations, suggestions for best practices in structuring courses, and emphasis on how the presentation of/representation in course reading lists is of the utmost importance (Forensic Anthropology Community 2021). The Forensic Anthropology Community (2021) website also provides training, research, and funding opportunities as well as a wealth of literature on integral topics outside of the traditional academic focus, including decolonization, pedagogy on difficult topics, deconstructing typology, ethics/professionalism, treatment of remains, harassment and discrimination, as well as training, accreditation, and certification. These are areas of critical exposure for education and practice in forensic anthropology that are often absent from the classroom or traditional training.

With continually teaching theories and methods developed or informed by scholars who are demonstrably racist, sexist, and imperial (Blakey 1987, 1999; Jackson 2001; Turner et al. 2006; Monteiro 2023), a prioritization of specific types of perspectives is continually communicated. While critical contextualization of these figures and their works is important and necessary, it is but one aspect of working to confront barriers in forensic anthropology education. In wanting to better cite and attend to the work and legacies of scholars who have been and continue to actionably address harm in forensic practice, we need to broaden the typical citational range of the subdiscipline. Scholars like Bond Day provide models for how to critically engage historical and vetted forensic anthropology techniques with a socially conscious and culturally aware lens/framework to critically engage with how people/remains are classified and how these classifications inform how people are understood and qualified (Curwood 2012). By primarily engaging with a disciplinary history that does not adequately credit or attend to a diverse array of scholars who have always been present within the field, syllabi and curriculum are used to further exclusionary conceptions of and within forensic anthropology.

### 1.2 Prioritizing Scholars Who Are Writing About and Practicing Culturally Informed Techniques and Who Engage With Discourse Around Shifting Paradigms

Tangible and visible inclusion matters in curating spaces to be more welcoming and to foster a sense of retained belonging (Winburn et al. 2021). While there may be limited subdisciplinary standardization in regard to forensic anthropology education, there is a pattern of commonly used and dominantly cited resources used to teach forensic anthropology (Blau 2018; DiGangi and Bethard 2021). Expanding this available bank of resources and adding a more diverse array of readings to syllabi/course curricula may work to communicate that diverse perspectives are primarily valued within forensic anthropology education. By demonstrably including and supporting diverse scholarship, it may also allow for the notion of “living” knowledge within forensic anthropology that is dynamic and engaged with changing contexts. Several scholars, both historical and contemporary, engage in work that actionably and actively challenges and builds up common practice. Referring back to scholars like Day and Hooton (1932) and Blakey (1987, 1998, 1999) and then looking forward to scholars like Lasisi (2021), discussions of race and ancestry assessment have been continually adapted to better address how cultural constructs are informed by increased knowledge about human evolution, awareness of history, and changing social context(s). Another example of representation and shifting paradigms in the field is brought to light by Tallman et al. (2022) in their discussion of the role of transgender bodies and forensic anthropological casework and research. Their survey highlights that forensic anthropological practitioners openly welcome an expansion of trans‐related research as a mechanism to expand the assumed sex binary in existing methodology and expose practitioners to a better understanding of gender‐affirming procedures that may be exposed in casework (Tallman et al. 2022).

Innovating, adapting, and challenging educational standards may also include shifts in teaching methods, meant to diversify and make more inclusive learning spaces. As discussed in a later section, one way to do this is to utilize reflective and reflexive writing practices in forensic education and training. Many subfields within anthropology have embraced personalized and nontraditional forms of writing—looking at narrative, speculative fiction, auto‐ethnography, and so on—and these works have helped contribute to expanded intellectual and theoretical engagement (De Smedt and De Cruz 2015; McKittrick 2021; Mackinlay 2022). Demonstrated engagement through action is important in communicating tangible attention to diverse thoughts and experiences. Purposefully seeking out scholars of diverse, nontraditional, and unique backgrounds may help to promote forensic anthropology as a more inclusive space and expand knowledge paradigms beyond limiting frameworks of singularity and objectivity.

### 1.3 Recruiting and Supporting Students, Scholars, and Professionals With Diverse Backgrounds and Interests to Purposefully Create Community That Engages With (Visible and Invisible) Diversity

Intellectual and theoretical diversity are informed by the realities of identity and positionality. It is important to consider how people are represented and what a diverse space can and should entail. Identities, vulnerabilities, and vantages are dynamic and intersecting, and so diverse spaces should attend to the complex and complicated realities of lived experience. In addition to having a diverse array of resources and educational schemes that promote and cite the perspectives of diverse scholarship, it is also important to promote a diverse and inclusive environment. In wanting to look toward a disciplinary future that does its best to ameliorate exclusion and ostracization, it is important to be purposefully inclusive of diversity (in thought, in positionality, in background, etc.) at all levels—looking to historical scholars, citation practices, leadership and administration, practice, teaching, and learning. In connecting diversity to ideological shifts in forensic frameworks, working to establish more interdisciplinary/transdisciplinary spaces may encourage a greater array of theoretical and training backgrounds within forensic programs. Understanding the unique position and responsibility of forensic anthropology, along with the intention of its methods, engaging with other methods, frameworks, and disciplines is not meant to diminish histories or standards of work. By curating educational, and thus professional, spaces to include and support a more diverse array of people and perspectives, there then becomes an opportunity to build on and continually apply both theory and practice. Having a subdiscipline that is committed to shifting, changing, and learning with people may inform and encourage long‐term commitment from the people within and around it.

To work toward actionable and inclusive change, we need to develop community‐based ethical guidelines that go beyond traditional IRBs and professional codes by co‐creating ethical standards with affected communities. We need to be transparent in our methods and their limitations by clearly communicating what forensic anthropology can and cannot do, especially in legal or human rights contexts (e.g., Plemons and Spiros 2025). We need to create ongoing and open channels for communities to express concerns, critique processes, and guide future work. In the classroom, we need to integrate anti‐racist and decolonial frameworks by embedding these into every level of training, not as add‐ons, but as foundational elements. Instructors need to advocate for and implement community‐grounded training that requires students to learn with and from communities—not just in classrooms or labs, but in real‐world, respectful collaborations (Adams et al. 2024). Finally, we need to address the history of harm by including curricula on the discipline's role in colonialism, systemic racism, and harm done in the name of science.

Mentorship can also be an integral road to inclusion and belonging (Tallman et al. 2022; Goliath et al. 2023). Mentorship can take many forms outside of the traditional and imperial “top‐down” approach. As a form of “social exchange” (Winburn et al. 2021, 115), mentors and social support can and should move beyond imparting solely academic insight; fostering socioemotional support as well as practical components of navigating educational spaces. As we begin to broaden our vision of educational landscapes, we will not only better serve ourselves, but pave the way for future students entering into forensic anthropology spaces that directly see themselves through mentorship, which serves as a mirror to their identity—an aspect cited as strong components to successful mentorship and academic success (Davis 2007; Brunsma et al. 2016; Bhalla 2019; Winburn et al. 2021; Goliath et al. 2023).

## 2 Changing Forensic Anthropology Epistemologies and Frameworks

In addition to more material, relational, and stepwise change, there are important theoretical and epistemological shifts that need to be considered when thinking about the future of accessibility, diversity, and equity within forensic anthropology. The following sections detail areas relevant to epistemology that are spaces for better attendance to and care for diversity in perspective. Both of the core discussions in this section engage with shifting paradigms and frameworks in how people think about and apply theory. In connection to the above discussion, both of these frameworks for shifting epistemology connect to and inform how curricula can be restructured, the inclusion of a more diverse array of scholarship, and attending to the well‐being of students and practitioners within the subfield.

### 2.1 Theoretical Reconsideration of “Objectivity” and Reliability

Science is often dominantly framed as a form of objective study. This perspective is true across many subjects and disciplines, including forensic anthropology and related fields (Winburn 2018; Clemmons and Winburn 2021). Science, as a dominant framework and as a function of positivism, emphasizes and relies on constructs of objectivity, testability, and reliability, which all allow knowledge to be “discoverable” (Bernard 2017). Objectivity frames scientific practice as removed, or striving toward removal, from human bias and imposes the notion of a correct understanding, removed from and beyond human/individual intervention (Deloria 1997). This construction of scientific knowledge, as aiming toward an objective truth, has allowed the subfield of forensic anthropology to conceptualize itself as atheoretical (DiGangi and Bethard 2021; Clemmons and Winburn 2021; Ross and Pilloud 2021). While the idea of forensic assessment as unbiased or removed from constructed context(s) has changed over time, as understanding around identity and as scientific techniques and principles have changed, a reconsideration of forensic anthropology as apolitical has been slower to shift.

Engaging with the concept of the gaze (Oldman 1975), we can argue that a person's specific vantage point informs what they value, what they consider, and how they work. The gaze, as “a dominant mode of looking” (S. M. Smith 2014, 2), is a manifestation of privilege, existing relative to positioning within institutions and other structures of power, impacting what people consider and what they hold as significant (Scott 1991; Césaire 2000). The idea here is that the nature of visual engagement from a specific vantage point imposes our own individual understanding and conceptualization (Scott 1991; S. M. Smith 2014). How we come to know (how we come to formulate “a gaze”) is informed by our ability to look at (the social performance of sight, the active gesture in the realm of visuality) and see (conscious perception) the world around us (S. M. Smith 2014, 2), which are mediated through things like education, positionality, background, and so on. Applying this to forensic anthropology education, the notion of the gaze—as informed by experience and bias—challenges forensic technique as removed from personal experience, vantage, or politics. Schemes of categorization for constructs like sex/gender and race/ancestry that are held as partially determining a person's identity are thus informed and mitigated by personal histories and interpretations of notions of evidence (Nakhaeizadeh et al. 2014). Maintained paradigms, knowledge production, and meaning‐making within forensic education cannot be purely objective because of our placement(s) in and biased engagement(s) with the world (Akena 2012)—this is not an argument of biased data or sampling; though that is a relevant consideration, but rather an argument of culturally and positionally mediated perspectives within the practitioner. The relevance and significance in attending to the role of bias and vantage in practice is well demonstrated through contentions/arguments around the use and effectiveness of techniques like sex or ancestry estimation as assessment criteria that are increasingly understood to be informed by larger cultural and individual contexts, impacted by history, migration, technology, politics, and other changes across time and space (Harrison 1995; Adams and Pilloud 2021; Ross and Pilloud 2021).

Looking toward conceptual changes and arguments for methodological adjustment, Bethard and DiGangi (2020, 428) called for a moratorium on the use of macromorphoscopic cranial traits in ancestry estimation and a greater focus on broader social issues surrounding the estimation of ancestry, pointing to the presence and impact of biased and socially informed phenomena like “missing white woman syndrome,” which serves as one of many tangible demonstrations of practice is mediated through cultural/social values. In response to and engaging with Bethard and DiGangi (2020) (based on counts of ‘cited by’ articles), there has been a significant amount of discursive writing and research with various points and perspectives. In direct relevance to the core argument (that ancestry estimation, as a standard practice, confers histories and realities of racialized/racializing violence), there have been additional and supportive arguments that highlight the role of scientific racism in the grounding and developing of the technique (Lasisi 2021; Ross and Williams 2021; Clarke 2022), the role and reach of terminology (Tallman, Kincer, et al. 2021; Tallman, Parr, et al. 2021; Martinez 2023), the significance of applying culturally informed frameworks (Gruenthal‐Rankin et al. 2023; Cunningham 2024), and the important of continually improving/building up past strategies with theory, technique, and information that more accurately and holistically account for natural human variety (Edgar and Pilloud 2021; Ross and Pilloud 2021; Winburn and Clemmons 2021; Zuckerman et al. 2025). This discussion highlights how historic forensic practice has been informed by violent and racist histories that correlated natural physical variation to the inherent presence of different “types of people,” which have a contemporary legacy that informs/impacts how different people are valued based on these schemes.

In another key component of the arguments around ancestry estimation, Ross and Williams (2021, 10) note that the use of continental‐based ancestry does not account for the substantial diversity that exists either within or between continents, and how it “lends credence to existing power structures and socioeconomic inequalities.” Racial and ancestral categories cannot be removed from the historical contexts and power imbalances that made and worked to validate them. The authors encourage a greater focus on population affinity, which incorporates microevolutionary and cultural factors (Ross and Williams 2021), which works to validate and emphasize how people and populations change over time, detaching from a static understanding of how people come to be and understand themselves. Similar to the discussion around the relevance of culture in conceptualizations of forensic race and ancestry, the discourse around the assessment of sex and gender, as dominant cultural constructions and understandings have changed (and continue to change) to better appreciate how neither exists as a binary nor as inherently determinable through discrete components of the body. Flaherty et al. (2023) define and scope this argument around the limiting nature of current sex estimation within the forensic profile, highlighting how assessments of sex should be better qualified/accompanied by a disclaimer that emphasizes that the forensic profile assesses from assigned sex at birth (ASAB), which does not necessarily or inherently match with the sex or gender identity of an individual. Engaging with a limiting binary within sex estimation does not account for the actual genetic, hormonal, or somatic diversity that informs people's sex expression and does not attend to how sex and gender are dynamic, non‐homogeneous identity facets.

Continuing these conversations within forensic anthropology education spaces by incorporating a greater diversity of voices and perspectives can continue to broaden and diversify theoretical perspectives within the discipline and highlight the dynamic nature of forensic anthropology. Highlighting past and present scholarship that works to confront schemes of harm and exclusion, like the work of Ross and Williams (2021), Flaherty et al. (2023), Blakey (1999), Go et al. (2021), Lasisi (2021), and so many others, helps to create a space that demonstrates its commitment to inclusion and belonging. Constructing the gaze as the dynamic interaction of perception and being is essential in considering how forensic anthropology (in education and practice education) is deeply entwined with the person doing the work. In understanding what the gaze is and how it functions as an instrument of power, defining its components may allow for the distinction between objectivity and subjectivity to be concretized and critically applied in education.

Upholding the idea of objective science promotes a singular worldview (Akena 2012; Winburn and Clemmons 2021). The notion of a scientific gaze within forensic anthropology, as taught to students, communicates a singular, “correct” paradigm of knowledge; the espousing of a perceived objective perspective or way of doing enforces the notion of an inherent and singular way to think about people, how to do things, and how to construct data (Akena 2012; McKittrick 2021). By framing forensic anthropology practice as scientific and apolitical in how it is approached, there is then the imposition of a way to know, a way to see. Highlighting how scientific objectivity (as a subdisciplinary value) is promoted by a specific theoretical paradigm that actively devalues other ways of knowing is meant to convey how it can function as an uncritical way to create barriers of separation between theory, history, context, and practice (Césaire 2000; Held 2019; D'Ignazio and Klein 2020; Clemmons and Winburn 2021). Forensic anthropology being framed as largely removed from politics and individual understanding/conceptualizations, works to distance the subdiscipline from the role and significance of dynamic anthropological theory, personal experience, unique positionality and vantage, and multiple ways of knowing in the material construction and perpetuation of culture (DeMarrais et al. 1996; M'Charek 2013; DiGangi and Bethard 2021). It also removes the reality of people (their intersectional contexts, backgrounds, priorities, biases, etc.) from the work, further isolating practice from dynamic, developing theory and the people who engage in that project, contemporarily and throughout time. If one way of knowing (which is informed by theory around and standards of harm and exclusion) is what is taught and then upheld, spaces for inclusivity and diversity are not able to be fostered (Akena 2012; McKittrick 2021; Chaussée et al. 2022). In wanting to include diverse people and perspectives, creating and maintaining a space where they are heard, welcomed, valued, and come to belong is important. Upholding what has been established as objective and reliable science, as a narrow and colonially informed standard, promotes a limitation of theoretical engagement, which works to inform how people of varying backgrounds and interests are not valued (Césaire 2000). If what forensic anthropology is and how it is practiced appreciates and values a singular way of knowing, then who is welcome in the space (to learn and then to practice) is limited.

### 2.2 Centering Reflexivity and Moving Away From Principles of the Apolitical

A relevant consideration in needing to recognize and incorporate both ‘the personal’ and ‘the political’ within forensic anthropology education is thinking about how mental health and well‐being are tied to diversity, inclusion, and feelings of belonging; attention to people—students, scholars, and practitioners—as individuals whose lives and backgrounds impact their work can function as another mechanism for fostering engagement, validation, and belonging. Forensic practitioners show a high degree of burnout or stress and report inadequate mental health support in their individual organizations or the profession overall (Goldstein and Alesbury 2021; Jeanguenat and Dror 2018; Kelty and Gordon 2015). Forensic anthropology, both education and practice, has a high personal cost because of its general mission and content—dealing with death, often in mass, is challenging for many and is compounded by systems and structures that depersonalize and decenter the people within them. Additionally, there are practical/financial costs to both education and professionalization in the discipline due to things like field schools, internships, and fellowships, which can be/are somewhat inaccessible and therefore more so available to people/students/professionals of privileged positions (Pink et al. 2025). Greater recognition and focus on the individual will serve to ensure people of all backgrounds and positionalities are both supported and able to support themselves as they move through evolving educational phases. Promoting the subdiscipline or its methods as apolitical removes them from theoretical contexts and works to remove them from the lives of people. If work and practice, which are valorized and upheld in educational spaces, are done through avenues that cause or promote epistemological harm, then students and practitioners are at personal/individual risk. With a greater focus on the researcher, on the self, the field can grow to intentionally and inherently be more inclusive and sensitive to the needs of practitioners, our colleagues, students, and those we serve. Attention to the ways that people approach, manage, and show up in their work will serve to ensure individuals of all backgrounds and positionalities are better supported as they move through evolving educational and professional phases.

In thinking about the connection between held beliefs, common practices, and dominant frameworks and issues around diversity and inclusion (in the recruitment and retention of students and scholars), it is important to envision future education and practice that could better include and value a more diverse array of people. The larger discipline of anthropology, along with other social sciences and related disciplines, has been shifting to include and valorize a wider range of perspectives/vantages, theories, analyses, and methods. This is well demonstrated through an increasingly visible, real‐time engagement with theory and practice through reflective and conscious forms of writing. The utility of thinking about and incorporating more personal methods of writing, such as auto‐ethnography and narrative story‐telling, in the discussion of teaching forensic anthropology is the ability to offer more opportunities for grounded or “rooted” reflection and reflexivity (Mackinlay 2022, 117–118) in more traditionally/dominantly scientific spaces. While distinct from the more technical types of assessment and writing that are paramount within forensic anthropology (that are still essential to teach), incorporating other and more reflective forms of writing into teaching and learning environments may help foster more inclusive, aware, and open spaces where these issues of isolation, disconnection, mental health concerns, and exclusion can be better addressed.

Practically valorizing forms of writing and reflection outside of more ‘traditional’ scientific frameworks not only demonstrably creates space to care about nondominant perspectives and methods but also concretely ties students, scholars, and practitioners to their work. While not necessarily translatable into more rigid forensic profiles or analyses, personal and reflective writing may have more of a space in the discipline‐facing discussion of work experiences, theoretical frameworks/orientations, and analytical processes. It is important to consider how our personhood and identity exist in and relative to how we engage with science and scientific inquiry (McKittrick 2021). It is essential to substantially value writing about individual experience (from the side of the scholar and practitioner) as a valid and valuable intellectual product that should be incorporated into educational materials/resources, in addition to and complementary to technical training(s). This point is made by several Black, Brown, and Indigenous scholars who have actively worked to uniquely incorporate auto‐ethnographic and reflective writing into a diverse array of disciplines. Borrowing from scholars like Watkins (2019), McKittrick (2021), and Parbhoo‐Ebrahim and Fourie (2021), the centering of lived reality in intellectual and academic work is important to the unknowing and unmaking of established paradigms that enforce how people are stratified and how power is used to wield harm. While disciplinary traditions, histories, and contexts are unique, and while this method/framework has not been specifically applied within forensic anthropology, exploring how reflective and reflexive writing has been engaged with and implemented in other, similar disciplines may help construct a model through while to incorporate styles like auto‐ethnography in forensic anthropology education. In thinking about curating and engaging with intellectual spaces, like classrooms, meetings and conferences, and interpersonal mentoring/advising, as inclusive, promoting lived experience and experiential knowledge as valuable. It is important to present and engage with both theory and practice as dynamic and personally impactful. Refusing to be removed from the work, particularly by marginalized scholars, is a way to counter standard systems of power in knowledge production (McKittrick 2021; Mackinlay 2022) and a way to actionably address how culture, context, and politics are relevant to forensic anthropology theory and practice (DiGangi and Bethard 2021; Ross and Pilloud 2021). In an educational space that is not already pedagogically standardized, being lively and dynamic in structuring how concepts are taught, what materials are used, and how theory is foregrounded seems like an accessible strategy for promoting more equitable, inclusive learning spaces (Love and Langley 2020; Pink et al. 2025). In wanting to move away from the limiting nature of positivistic science, informed by known legacies of harm and exclusion, centering the experiences of students and scholars through avenues like auto‐ethnography and reflective writing allows for the valuation of non‐technical education as also significant (Stoetzler and Yuval‐Davis 2002; McKittrick 2021). Encouraging critical engagement with theory and the act of reflective writing as standard practices in the forensic anthropology classroom may also connect to mitigating the burden of labor placed on faculty and scholars who engage with more critical texts and more applied theory when teaching and writing. If a theoretically connected and validating space is the educational norm, then the availability of resources and the burden of care to attend to diverse cohorts of students is more equitably distributed across institutions and educators.

## 3 What It May Mean to Change

These factors are but a few within the extensive discussion around systemic barriers to inclusion and diversity within academia, generally, and forensic anthropology, more specifically. Tackling both the experienced and felt barriers within this field and the premises that underlie them is important in making tangible progress toward belonging, equity, and well‐being within the subdiscipline. Looking toward the future of forensic anthropology and the larger discipline, working to acknowledge, address, and shift foundational tenets is important in wanting to mitigate and combat histories of harm. Upholding the mythology of objectivity, uncritically holding forensic work as atheoretical or apolitical, and limiting valued forms of theoretical engagement and work are examples of how dominant paradigms inform subdisciplinary structuring. This structuring, as it serves limiting ways of knowing and doing to uphold exclusive forms of violence, is important to critique and explore in wanting to promote spaces of inclusion and belonging. Systems of power have, and continue to, benefit from exclusivity. By naming and detailing tangible mitigating strategies, confronting exclusion's relationship to the exploitation and maintenance of vulnerabilities and barriers is relevant in looking at how ideological standards promote harm.

## Author Contributions

**Taylor S. Borgelt:** conceptualization (equal), writing – original draft (equal), writing – review and editing (equal). **Jesse R. Goliath:** conceptualization (equal), writing – original draft (equal), writing – review and editing (equal). **Erin B. Waxenbaum:** conceptualization (equal), writing – original draft (equal), writing – review and editing (equal).

## Data Availability

No new data were created or analyzed in this study. Data sharing is not applicable to this article.
